# The Effects of 24 weeks of Resistance Training with Simultaneous Elastic and Free Weight Loading on Muscular Performance of Novice Lifters

**DOI:** 10.2478/v10078-011-0043-8

**Published:** 2011-10-04

**Authors:** Todd C. Shoepe, David A. Ramirez, Robert J. Rovetti, David R. Kohler, Hawley C. Almstedt

**Affiliations:** 1 Human Performance Laboratory, Department of Healthand Human Sciences, Loyola Marymount University, Los Angeles, CA; 2 Human Performance Laboratory, Department of Mathematics, Loyola Marymount University, Los Angeles, CA

**Keywords:** variable resistance, isokinetic exercise, muscular performance, elastic band loading

## Abstract

The purpose of this investigation was to assess the effectiveness of variable resistance as provided through elastic plus free weight techniques in college aged males and females. Twenty novice lifters were randomly assigned to a traditional free weight only (6 males and 5 females) or elastic band plus free weight group (5 males and 5 females) and 9 more normally active controls (5 males and 4 females), were recruited to maintain normal activity for the duration of the study. No differences existed between control, free weight and elastic band at baseline for age, body height, body mass, body mass index, and body fat percentage. One-repetition maximums were performed for squat and bench press while both strength and power were assessed using isokinetic dynamometry. Elastic groups and free-weight groups completed 24 weeks of whole body, periodized, high intensity resistance (65–95% of one-repetition maximum) training three times/week. Training programs were identical except that the elastic group trained the barbell squat, bench press and stiff-legged deadlift with 20–35% of their total prescribed training loads coming from band resistance (assessed at the top of the range of motion) with the remainder from free weight resistance. A mixed-model analysis revealed that peak torque, average power and one-repetition maximums for squat were significantly greater after training for the elastic group compared to the control (p<0.05). In addition, the free weight group also showed significantly greater improvements over the control in peak torque and one-repetition maximums for squat and bench press. No significant differences were observed between the elastic band and free weight groups. Combined variable elastic band plus free weight exercises are effective at increasing strength and power similar to free-weights alone in novice college aged males and females. However, due to complexity in set-up and load assignment elastic adoption by novice lifters in an unsupervised situation is not advised.

## Introduction

In the perpetual endeavor to improve the efficiency of training routines, new methods are constantly implemented at all levels of strength and conditioning. In recent years, one of these that has gained widespread acceptance in training programs throughout the world is the combination of elastic bands (EB) added to free weight (FW) exercises (Baker & Newton, 2005; Findley, 2004; Simmons, 1996; 1999; Swinton et al., 2009; Warpeha, 2002). Despite common usage and anecdotal support, controlled prospective research has been slow to investigate the claim that this form of variable resistance exercise is an effective training technique for improving muscular strength and explosive power. Only recently have research findings begun to surface that support these practices (Anderson et al., 2008; Cronin et al., 2003; Ghigiarelli et al., 2009; Jakubiak & Saunders, 2008; Mccurdy et al., 2009; Rhea et al., 2009).

Arising from the sport of competitive powerlifting (Simmons, 1996; 1999), the addition of elastic bands to a traditional form of free weight resistance exercise is suggested to effectively alter the kinetics of multi-joint exercises such as the squat (Israetel et al., 2010; Neelly et al., 2010; Wallace et al., 2006). While some evidence does not support this hypothesis (Coker et al., 2006; Ebben & Jensen, 2002), the work of Wallace et al. (2006) demonstrated that if performed with maximal voluntary effort (Behm & Sale, 1993; Young & Bilby, 1993), elastic bands allow for higher forces and power outputs than free-weights alone during single bouts of squats. Further studies have suggested that force-velocity-power relationships are acutely altered throughout an entire range of motion on squats (Israetel et al., 2010) and bench press (Baker & Newton, 2009) by training with elastic bands.

Although additional work has recently shown that combined elastic plus free weight exercises in athletic populations over short durations (7–12 weeks) is effective at increasing strength (Anderson et al., 2008; Ghigiarelli et al., 2009; Mccurdy et al., 2009; Rhea et al., 2009) and power (Rhea et al., 2009), mixed results have been reported as to whether combined training is more effective than traditional training. The investigations into this question have found no group differences (Ghigiarelli et al., 2009; Mccurdy et al., 2009; Rhea et al., 2009) and significant group differences in strength development (Anderson et al., 2008), trends for group differences in power development (Ghigiarelli et al., 2009) and significant group differences in power outcomes (Anderson et al., 2008) when comparing EB to FW training. These authors frequently suggested trends and short duration of the exercise intervention as potentially limiting the ability to effectively discern true differences between FW and EB training methods. Furthermore, each of the previously published training studies was conducted in college athletes for short durations (Anderson et al., 2008; Ghigiarelli et al., 2009; Mccurdy et al., 2009; Rhea et al., 2009) and only one included a mixed participant pool of males and females (Anderson et al., 2008). While the work of Anderson et al.(2008) suggests benefits to trained athletic populations, we were further interested in elucidating the efficacy of these training modalities because they are commercially advertised and anecdotally utilized by novice lifters. The purpose of this study was therefore to assess the effectiveness of variable resistance techniques (as provided by combined elastic and free weight loading) to traditional free weight resistance only exercise in untrained, college aged males and females over a long duration.

## Methods

### Experimental Approach to the Problem

Following approval from the Institutional Review Board for the Protection of Human Subjects at Loyola Marymount University, 34 recreationally active males and females between the ages of 18–23 were recruited for participation in this study. Both sexes and a diverse mix of races were specifically included in a mixed-subjects pool in order to adhere to National Institutes of Health objectives of inclusion in prospective human research. Following a completion of a written, informed consent prior to beginning any phase of the study, 24 participants volunteered for random assignment into either an elastic band plus free weight group (EB; n=12) or a free-weight only group (FW; n=12). The remaining volunteers were assigned to a normally active control group (CON; n=10) and instructed to maintain their current lifestyle of physical activity for the duration of the intervention. Both FW and EB groups then performed 24 weeks of resistance training, three days per week at periodized intensities varying between 67–95% of 1RM on the multijoint exercises of bench press, squats and deadlifts (DL), and 67–80% of 1RM for seven additional upper and lower body assistance exercises. Because the intention of this study was to identify the effects of EB exercise on a contextualized, practical scenario of untrained collegiate students, the program was intentionally shaped around the academic calendar. In total, a 24-week macrocycle of training occurred in two 12-week mesocycles coinciding with the academic calendar of the host institution and were separated by a four-week layoff for winter holiday as well as a one-week interruption for spring break. While this provided an extended detraining time in the middle of the intervention, this modeled the likely behaviors of most college students and increases the generalizability of the findings to a broader population. Prior to the onset of training, all volunteers completed questionnaires to assess health history, physical activity, dietary intake, and menstrual history (females only) for use in prescreening and as part of another related research investigation. No participants reporting using anabolic steroids or dietary supplements (other than multivitamins) at baseline, 12 weeks and 24 weeks which would have represented exclusion or dismissal from the study. Baseline testing for demographics, anthropometrics, and isokinetics occurred one week prior to the onset of training, with 1RM values for squats (SQ) and bench press (BP) assessed after a two-week acclimatization phase to allow for technique familiarity.

### Participants

Potential participants of the study were selected from the Loyola Marymount University student body while interviews and pre-screening produced an equal representation of both genders in the CON (5 males and 5 females), FW (6 males and 6 females), and EB (6 males and 6 females). Study exclusion criteria included no experience with resistance training (past 12 months), no current musculoskeletal injuries limiting training, and a BMI between 18 and 30. Four exercisers (two male and two female) and one control (male) dropped out of the study after baseline testing for varying reasons including two males and one female who cited scheduling difficulties between training and academic responsibilities as being too great. An additional female ceased training in the third week due to the re-emergence of a previous back condition that became exacerbated by the exercise protocol. In total, throughout the study duration there are complete data sets for 9 members of the CON group (4 male and 5 female), 10 members of the FW group (5 male and 5 female), and 10 members of the EB group (5 male and 5 female). Table 1 displays baseline characteristics of participants, demonstrating no significant differences between groups.

### Procedures

#### Resistance Training Program

The training program was designed to be contemporary, high-demand, yet realistic for recreational collegians designed in part to promote muscular development, strength and power variables. The program was performed for 24 weeks with a frequency of three nonconsecutive days per week under the close supervision of a personal trainer to ensure correct technique, offer encouragement, ensure adherence and decrease chance of injury. Day one was designed to emphasize the lower body, day two the upper body, and day three a combined exercise day with the core musculature worked at the conclusion of each of the three training days. The program was periodized and included a two-week general training phase for the purposes of physical preparation, acclimatization, and technique instruction prior to the implementation of significant increases in intensity or load.

The subsequent 10 weeks were marked by an undulating periodization program where intensity on the multijoint exercises (e.g. BP, SQ, DL) were increased according to the guidelines for strength and power development as put-forth by the National Strength and Conditioning Association (NSCA) (Baechle & Earle, 2008). After the 12-week training period, volunteers were permitted a three week break for the winter holidays. A similar 2-week anatomical adaptations phase followed by 10 weeks of training, followed the break and coincided with the spring semester of classes.

**Figure 1:**
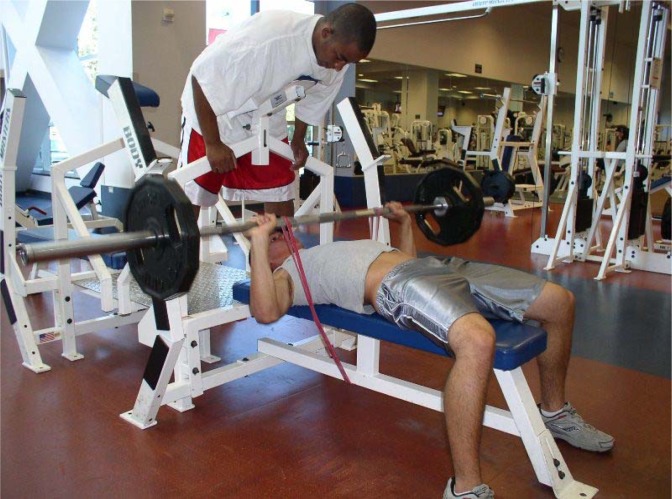


**Figure 2:**
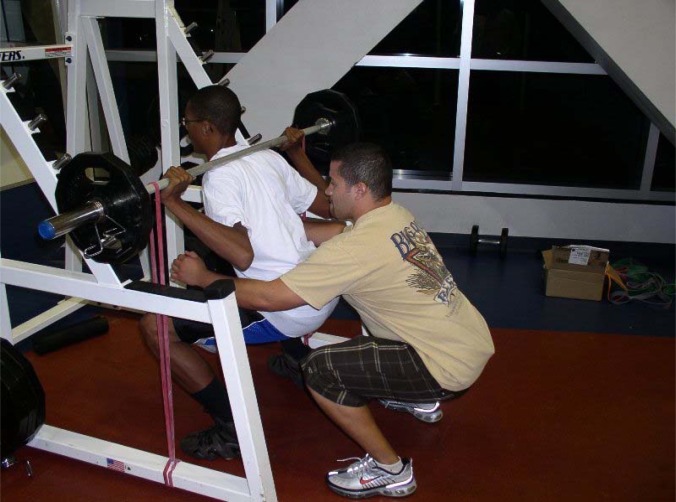


**Figure 3:**
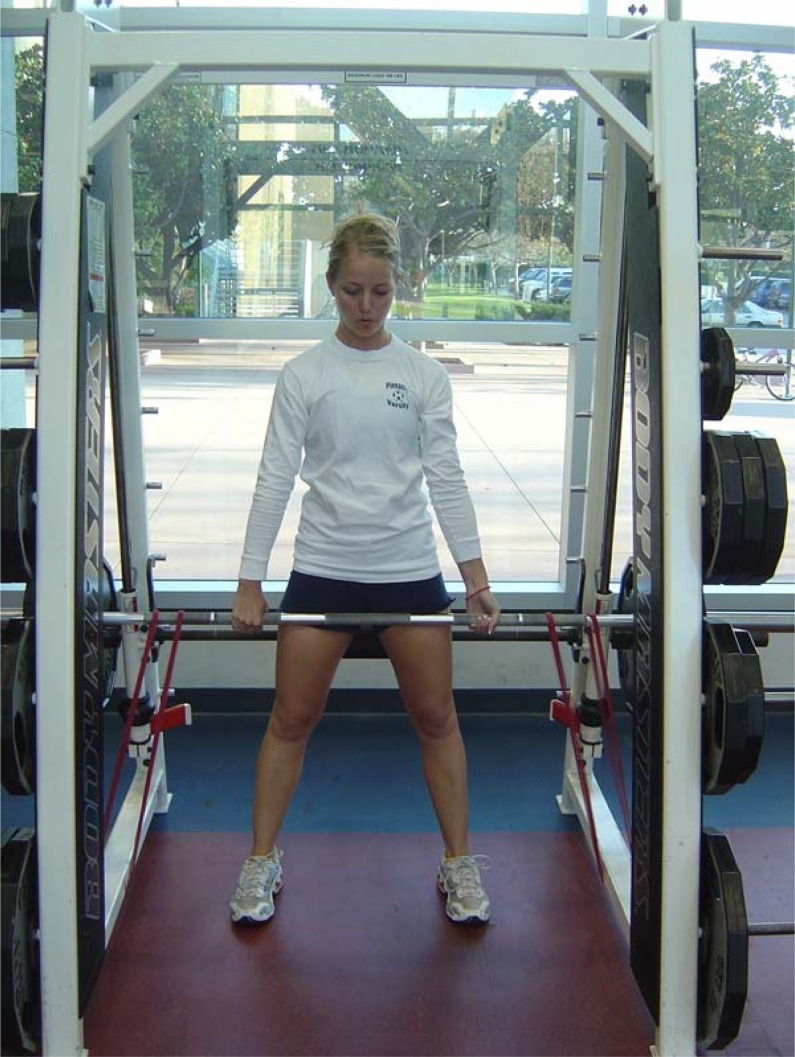


The program undulated on a daily basis in a non-continuously increasing fashion where the intensities varied from 67–95% of one repetition maximum. There were also heavy, medium, and light intensity days where the resistance was 100, 90 and 80%, respectively, of the assigned training intensity of that day (i.e. light day would be 80% of 85% of 1RM for six repetitions not performed to failure). Training loads were adjusted following every 1RM test and throughout the training program using a 2x2 rule, whereby if the participant was able to perform two or more repetitions over the prescribed number on the last set for two consecutive workouts, the load was increased on the subsequent workout. Sets, reps, and rest periods were adjusted according to the goal of the training day to reflect appropriate metabolic training and recovery. For example, on a given strength-focused day in week 11, multijoint exercises were performed to 4 sets of 6 repetitions at 85% intensity with 2 minutes rest between sets. Conversely, on a lower intensity day with 75% of 1RM loads, 3 sets of 10 repetitions were performed with less than 60 s rest in-between sets. With the exception of the SQ, BP, and DL where subjective velocity failure was used as a terminal criterion (e.g. when movement speed decreased sufficiently), all other exercises required spotter intervention for set conclusion. However, the last repetition where spotters provided aid was never counted. Each training session lasted about 75 minutes; beginning with a 10 minute cardiovascular general warm-up, followed by a specific warm-up of at least one preparatory set (< 50% 1RM) for each multijoint exercise, then 30 minutes of resistance training as described above. Each session concluded with 10 minutes of abdominal and flexibility training. All programming considerations were influenced by a desire to increase adherence and compliance with the training program while minimizing dropout rates. For this reason, abdominal and post workout flexibility training were included in the training program as well as additional exercises other than the primary three banded exercises (e.g. SQ, DL, and BP). Table 2 contains a complete list of exercises in the order that they were performed each training day. In total, retention rates (85%) and adherence for this volunteer research study were both high for a 24-week investigation with 1354 sessions completed from the prescribed 1441 (after adjustments for dropouts) for a total adherence of 94%. Participants in the EB and FW groups adhered to identical training protocols with the only exceptions being the loading application on BP (Figure 1), SQ (Figure 2), and stiff-legged deadlift (Figure 3) exercises with instructions given to the EB group who were asked to perform each concentric phase of the elastic exercise with maximal voluntary effort.

For both training groups and all exercises, every eccentric contraction was to last three seconds with the concentric contractions occurring for two seconds with the only exception being the EB concentric contraction. Citing the work of Wallace et al. (2006) who demonstrated that differences in power between FW and EB exercise were reduced when the total load coming from elastic resistance as assessed at the lock-out-phase of each exercise exceeds a threshold of 35%, all band loads were kept within a zone of 20–35% of the total resistance.

Individualized, programmed Excel spreadsheets were created for each participant for each banded exercise that automatically populated the cells according to the differences in height, arm length, and 1RM of each participant. Regression equations were generated in order to correctly identify the relative contribution to load of each band (Shoepe et al., 2010) and bands of varying thicknesses were identified according to a color-coding system on the spreadsheet to ensure that intensity for every set and each participant was accurate to the program specifications. In total, the principal investigator needed to only input lockout height for DL, BP, and SQ along with 1RM and each cell of the spreadsheet would populate with the amount of additional weight to be placed on the bar while the color of the cell would indicate the appropriate band.

#### Performance Testing

Isokinetic testing of the quadriceps during concentric extension was completed with a dynamometer controller (BIODEX model 900-350, Shirley, New York, USA.) at speeds of 30, 90, 150, 210, 270, and 330 degrees per second. Prior to testing, participants performed five minutes of light cardiovascular activity on a bicycle ergometer before being placed in a seated position on the ergometer with restraints placed across the shoulder, waist, and mid-thigh. The lever pad was positioned on the posterior tibia with the most inferior edge of the pad two cm from the lateral malleolus. Testing began in serial progression beginning with the fastest velocity in sets of five contractions where the participant was encouraged to contract with maximal effort throughout the entire range of motion from approximately 90 degrees of flexion to full extension. Peak torque for each test velocity was determined as the highest torque achieved during the set of five repetitions at each velocity. Average power was calculated as the product of the measured torque values described previously and the respective test velocity occurring across all five repetitions. All isokinetic final post testing was completed 3–5 days after the last training session of the second 12 weeks to allow for adequate supercompensation and recovery from the training sessions.

Testing of one repetition maximum (1 RM) occurred in week 3, 12, and 24 according to protocols set by the NSCA (Baechle & Earle, 2008) for the SQ and BP exercises. Strength values were established using the 1RM test completed in week 3, after two weeks to acclimatize to the exercise protocol. The strength values measured during week 3 were used to set the initial loads for the program. The use of knee or wrist wraps, squat suits, and weight belts were prohibited from every aspect of the training program and testing protocols.

### Body Composition

Seven-site skinfold procedures (Jackson & Pollock, 1978; Jackson et al., 1980) were used with Lange calipers (Beta Technology, Santa Cruz, CA) to determine body density, then percent body fat was estimated using the Siri equation (Siri, 1956).

### Statistical Analysis

All data were analyzed as “absolute change from baseline” by subtracting the pre-study value from the Week 24 value.

For the extension peak torque and extension average power measures, the study constituted an unbalanced mixed-effects repeated-measures design with treatment as the between-subject factor and angular speed as the within-subject (repeated) factor. Gender was not included as a factor as its potential effects were largely removed by baseline-correction. The MIXED procedure in SAS was used with an unstructured (generalized) covariance matrix for the repeated measure, and with subjects as a random effect nested within treatment group. Main effects were assessed using the Type-III test of fixed effects. Post-hoc analyses were conducted using the Tukey-Kramer adjustment for multiple pairwise comparisons. For purposes of data presentation (but not for statistical analysis), an “integrated” value for each measure, taken as the average over all angular speeds, was also calculated.

For the bench press and squat measures, the data were analyzed (separately for each measure) using an unbalanced one-way fixed-effect design with treatment as the fixed (between-subject) factor, also with the MIXED procedure in SAS (SAS Institute Inc., Cary, NC, USA) as described above.

## Results

### Anthropometrics

There were no observed differences between groups at baseline for height, weight, BMI, or body fat percentage. There were likewise no differences seen after 24 weeks in time, between group, or group x time interactions seen for height, weight, BMI, or body fat percentage.

### Isokinetic torque

**Figure 4:**
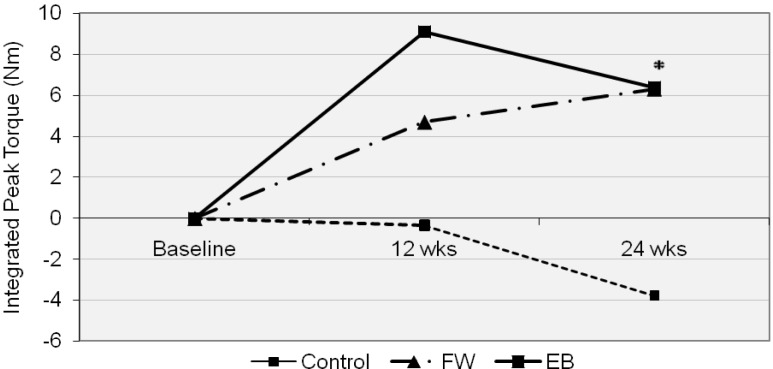
Values are presented as percent change from baseline to 24 weeks. * denotes statistically different from CON (p < 0.05). CON=control group; FW=free weight group; EB=elastic band and free weight combined training group

Baseline and 24 week isokinetic torque data are displayed in Table 3. Mean (±SD) change from baseline in the integrated peak torque was −5.1 (11.7), 8.6 (6.8), and 8.7 (12.6) N x m for the CON, FW, and EB groups, respectively. There was a significant overall treatment effect (p = 0.013); post-hoc analysis confirmed that both the FW (p = 0.025) and EB groups (p = 0.024) differed from the CON group but did not significantly differ from each other. This integrated peak torque data can be seen in Figure 4.

### Isokinetic average power

**Figure 6:**
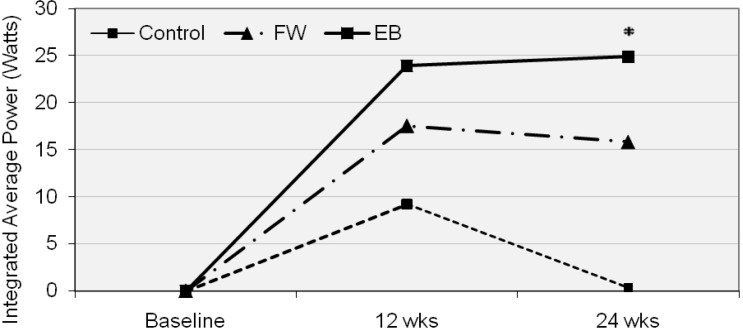
Values are presented as the integrated average power values encompassing all speeds at baseline, 12 and 24 weeks. * denotes statistically different from CON (p < 0.05). CON=control group; FW=free weight group; EB=elastic band and free weight combined training group

The average power data from baseline and 24 weeks are shown in Table 4. Mean (±SD) change from baseline in the integrated average power was 0.4 (13.5), 15.8 (19.0), and 24.9 (27.0) W for the CON, FW, and EB groups, respectively. There was a significant overall treatment effect (p = 0.017); post-hoc analysis revealed that the EB group significantly differed from the control group (p = 0.013), but the FW group did not differ from the control group. This integrated average power data can be seen in Figure 6.

### One-repetition maximums

Multijoint 1RM strength data are presented in Table 5. For the 1-RM bench press, mean (SD) change from baseline was 0.0 (4.8), 10.2 (6.2), and 5.7 (4.8) kg for the CON, FW, and EB groups, respectively. There was a significant overall treatment effect (p = 0.001); upon post-hoc analysis, the FW group significantly differed from the control group (p <= 0.0008), but the EB group differed only marginally (p = 0.071) from the control group. The two exercise groups did not significantly differ from one another.

For the 1-RM squat, mean (SD) change from baseline was 7.6 (13.3), 21.9 (10.6), and 22.0 (12.8) kg for the CON, FW, and EB groups, respectively. There was a significant overall treatment effect (p = 0.027); post-hoc analysis showed that the EB group significantly differed from the control group (p = 0.043), and the FW group nearly so (p = 0.051). Again, the two exercise groups did not significantly differ from one another.

## Discussion

An original contribution of this study is the finding that EB has been shown to be effective at significantly increasing isokinetic strength when taken as a whole, across a spectrum of velocities (Figure 6). Individualized independent analysis of the test velocities did not yield significant findings except at the highest test speed. When an integrated approach was included that allowed for a single analysis of difference across all test velocities, the EB group was shown to be effective at increasing isokinetic torque. The present data is nonetheless in agreement with long accepted principle of specificity of adaptation to training forces (Pereira & Gomes, 2003) and can be explained through the work of Israetel et al. (2010) who described the differences in force and velocity throughout an entire range of motion during maximal voluntary effort contractions of the type performed by the EB. In essence, greater forces are generated during each banded repetition during the first half of the eccentric and last half of each concentric contraction due to the decreasing overall load from shorter band length on the way down and increasing lengthresulting in higher overall loads on the way up (Israetel et al., 2010). This creates a variable-resistance exercise that allows one to carry momentum and enhanced muscular activation into the completion of each repetition. This might allow the lifter to overcome larger forces over the last portions of the concentric extension that is more in parallel with the joint kinematics of the lower extremity. Higher velocity movements during performance testing have previously been reported with other investigations of variable resistance loading (Baker & Newton, 2009).

Thus, with a greater exercising force production in the muscle during all training with the EB squat, which incorporates to a great extent the knee extensors, it is reasonable to assume greater adaptation and strength development during isokinetic testing. Average integrated power was also shown to increase in EB but not in FW across all test velocities taken as a whole even though the training groups were not statistically different from one another (Figures 6 and 8). At least two other studies have demonstrated increases in power generation in the lower body following EB training (Anderson et al., 2008; Rhea et al., 2009) that appear to be in agreement with these findings. Anderson et al. (2008) showed an increase in peak power of 4.5% after 7 weeks, while Rhea et al. (2009) reported an increase of 18% in peak power after 12 weeks of EB squat training as calculated from counter movement jumps. The integrated power increase of 25% coupled with an increase in average power of 32% in the EB group of this study at the highest test velocity is reasonable in comparison due to a much longer time frame and again, the novice training status of these participants.

The combination of multijoint, closed-kinetic chain and singlejoint, open-kinetic chain activities adds to the strength of this investigation. Muscular adaptations due to resistance training are specific to the type of training the muscle is subjected to with discrepancies found when the training protocol and testing modality differ (Rutherford et al., 1989).

In this study, the participants performed the singlejointed leg extension and the multijointed squat exercises during both the training and testing sessions. The majority of performance improvements in short term training interventions have been attributed to neurological improvements associated with increased agonist activation, decreased antagonist activation and muscular coordination (Carolan & Cafarelli, 1992; Hakkinen et al., 1985; Hakkinen et al., 1988; Moritani & DeVries, 1979). It is further understood that more complex movements such as the squat require greater neurological learning than singlejoint isolation activities such as the leg extension and that slight improvements in performance are likely to be seen over time in control group despite not participating in the training sessions. Similar to the Anderson et al., (2008) study of EB training in athletes, neither of our training groups demonstrated improvements in lean body mass. With no significant differences in body composition, neural mechanisms are likely playing the dominant role in performance improvements seen in both training groups- an expected finding with novice lifters. The foreign loading pattern of the EB group was anecdotally confirmed by participants who commented on an unfamiliar feeling of the resistance during 1RM testing, which could have decreased performance in these assessments. However, with confirmatory evidence provided by isokinetic testing, the performance improvements seen with the EB group reduce the suggestion of Type I error in this study.

Anecdotal suggestion has for years purported hypothesized benefits in muscular performance associated with elastic and chain loaded variable resistance exercise (Baker & Newton, 2005; Berning & Adams, 2004; Findley, 2004; Simmons, 1996, 1999; Warpeha, 2002). Only in recent years is evidence now accumulating to support the advocacy of variable resistance training techniques for the development of muscular strength and power. However, this is the first study to demonstrate the effectiveness of these techniques in novice male and female lifters. The hypothesized benefit of this training method is twofold. First, maximal torque production of the human skeletal system is not constant. In fact, it varies throughout a given range of motion (Cabri, 1991) and by matching the loading pattern to naturally occurring leverage, a greater overload of the muscular system might ensue which would promote greater gains in muscular performance. Second, variable resistance of the type investigated here allows for the use of maximal effort contractions, which have been shown to be more effective than submaximal effort training (Jones et al., 1999; 2001). Another such methodology in common use results in airborne phases as seen in jump squat training (Baker et al., 2001; Mcbride et al., 2002). In contrast to jump squats, partial elastic and chain loading could possibly produce similar specificity and benefit with reduced injury potential through avoidance of the heavy compressive impact forces encountered with the eccentric loading following airborne activities.

### Practical Applications

Combined elastic band and free weight exercise is a training method gaining in frequency and application in strength and conditioning of both novice and high performance athletics. These data suggest that variable resistance exercise created through the application of elastic bands in combination with free-weights performed to maximal voluntary effort is effective at improving muscular performance variables. Furthermore, this study found no group differences between FW and EB resistance training benefits after 24 weeks of periodized training suggesting that EB is a suitable alternative to traditional methods in novice, recreationally active collegiate males and females respectively.

At present, the increasing body of literature suggests that for both novice and experienced individuals, EB exercise can provide benefits in strength and power at least in equivalence to that of FW alone. However, one of the most important findings of this study is that elastic band set-up is challenging and load assignment is extremely complicated. With no obvious advantage shown in this EB training program in comparison to FW in novice lifters, unsupervised and broad recommendation does not seem warranted in novice lifters. This study, in conjunction with the work of Anderson et al. (2008) who demonstrated significantly higher increases in BP and SQ 1RM in well-trained athletes with the absence of muscular hypertrophy suggests that neurological improvements due to EB training can be very beneficial in athletic populations where it could be used to stimulate renewed adaptation during training plateaus. It is recommended that strength and conditioning professionals consider the status of the participant and the possible level of supervision when adopting variable resistance activities, utilizing combined elastic and free weight loading for multi-joint exercises, in conjunction with a well-rounded traditional free weight program targeted for the development of muscular strength and power as part of a comprehensive training program macrocycle.

## Figures and Tables

**Figure 5:**
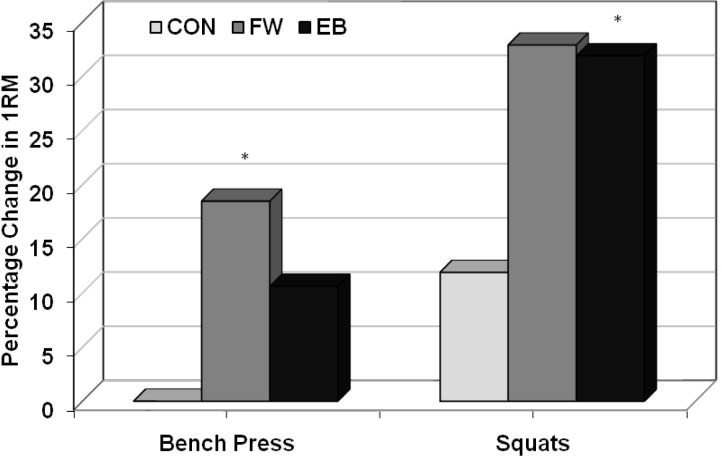
Values are presented as the integrated peak torque values encompassing all speeds at baseline, 12 and 24 weeks. * denotes statistically different from CON (p < 0.05). (Here, the single asterisk (*) denotes EB different from CON as well as FW differences from CON.) CON=control group; FW=free weight group; EB=elastic band and free weight combined training group

**Table 1:** Baseline and Post 24 week Anthropometrics

Group	Body Height (cm)	Body Mass (kg)	Age (yrs)	Body Fat (%)	BMI (kg/m^2^)
CON (n=9)					
PRE	165.5 ± 11.7	67.6 ± 16.3	19.4 ± 1.4	21.9 ± 10.0	23.8 ± 3.0
POST	164.9 ± 12.0	67.4 ± 14.3	20.1 ± 1.4	23.0 ± 9.9	24.6 ± 3.3
FW (n=10)					
PRE	169.7 ± 9.7	64.6 ± 9.0	19.9 ± 1.2	18.7 ± 8.2	22.4 ± 2.0
POST	169.8 ± 10.1	66.5 ± 8.2	20.6 ± 1.2	18.9 ± 10.1	23.1 ± 1.9
EB (n=10)					
PRE	171.1 ± 9.5	68.2 ± 8.0	20.0 ± 1.4	19.5 ± 10.9	23.3 ± 2.1
POST	171.3 ± 9.5	68.9 ± 9.5	20.7 ± 1.4	19.1 ± 9.4	23.4 ± 2.3

Values are presented as means ± Standard Deviation (SD).

No differences were noticed at baseline or after 24 weeks for any between or within groups variable (p > 0.05).

CON=control group; FW=free weight group; EB=elastic band and free weight combined training group

**Table 2:** Training Program

Workout 1	Workout 2	Workout 3
Squat^*^	Bench press^*^	Squat^*^
Leg extension	Seated row	Bench press^*^
Stiff-legged deadlift^*^	Standing dumbbell press	Stiff-legged deadlift^*^
Seated heel raise	Standing barbell curl	Seated rows
Planks (side and front)	French press	Plank and crunch
	Shoulder shrug	
	Abdominal crunch	

* Denotes an exercise that was banded in the EB group

**Table 3:** Isokinetic Knee Extension Peak Torque at Baseline and 24 Weeks

Velocity (degrees/s)	**CON**		**FW**		**EB**	

	PRE (Nm)	POST (Nm)	PRE (Nm)	POST (Nm)	PRE (Nm)	POST (Nm)
30	159.7 ± 46.3	151.2 ± 48.1	168.3 ± 40.7	172.0 ± 41.3	182.6 ± 39.7	181.2 ± 36.8
90	146.6 ± 43.9	129.9 ± 41.0	145.2 ± 42.1	153.6 ± 40.6	154.7 ± 36.2	156.5 ± 37.2
150	123.2± 42.1	114.6 ± 35.5	121.3 ± 35.4	130.1 ± 36.3	132.7 ± 33.7	139.4 ± 35.8
210	105.2 ± 35.9	104.2 ± 34.2	107.9 ± 33.8	114.7 ± 32.2	113.7 ± 31.0	128.1 ± 33.2
270	96.3 ± 34.5	93.9 ± 31.1	91.9 ± 29.4	102.6 ± 29.5	101.0 ± 27.5	114.8 ± 30.3
330	82.6 ± 28.3	85.0 ± 28.2	78.6 ± 26.8	91.5 ± 25.7	83.2 ± 26.4	100.1 ± 23.4

Values are presented as means ± SD; CON=control group FW=free weight group; EB=elastic band and free weight combined training group

**Table 4:** Isokinetic Knee Extension Average Power at Baseline and 24 Weeks

	**CON**		**FW**		**EB**	
	
Velocity (degrees/s)	PRE (Watts)	POST (Watts)	PRE (Watts)	POST (Watts)	PRE (Watts)	POST (Watts)
30	48.2 ± 17.2	43.2 ± 14.4	52.0 ± 10.9	51.5 ± 14.3	52.1 ± 10.7	54.6 ± 14.3
90	124.0 ± 43.3	115.0 ± 38.6	129.9 ± 36.6	131.4 ± 33.5	143.1 ± 38.6	144.5 ± 35.9
150	174.4 ± 56.2	167.7 ± 55.5	178.9 ± 53.5	185.9 ± 48.5	201.1 ± 58.6	203.3 ± 50.0
210	196.9 ± 74.8	202.6 ± 66.4	208.6 ± 67.9	218.0 ± 58.2	225.7 ± 77.3	258.1 ± 69.3
270	217.4 ± 80.1	222.3 ± 74.8	206.7 ± 62.9	240.3 ± 72.1	235.4 ± 75.1	283.7 ± 70.1
330	194.0 ± 72.7	206.4 ± 70.0	177.2 ± 61.7	221.2 ± 52.1	192.4 ± 76.2	254.8 ± 58.2

Values are presented as means ± SD.

CON=control group; FW=free weight group EB=elastic band and free weight combined training group

**Table 5:** Isotonic Strength as Assessed with One-Repetition Maximums at Baseline and 24 Weeks

	**CON**		**FW**		**EB**	
	
Exercise	PRE (kg)	POST (kg)	PRE (kg)	POST (kg)	PRE (kg)	POST (kg)
Bench Press	53.5 ± 29.3	53.5 ± 26.6	56.3 ± 30.3	66.7 ± 27.0	53.6 ± 21.0	59.3 ± 24.5
Squats	63.9 ± 27.2	71.5 ± 25.3	66.9 ± 16.5	88.9 ± 23.2	69.3 ± 27.0	91.4 ± 31.9

Values are presented as means ± SD.

CON=control group; FW=free weight group; EB=elastic band and free weight combined training group
